# A Temporal Examination of Platelet Counts as a Predictor of Prognosis in Lung, Prostate, and Colon Cancer Patients

**DOI:** 10.1038/s41598-018-25019-1

**Published:** 2018-04-26

**Authors:** Joanna L. Sylman, Hunter B. Boyce, Annachiara Mitrugno, Garth W. Tormoen, I-Chun Thomas, Todd H. Wagner, Jennifer S. Lee, John T. Leppert, Owen J. T. McCarty, Parag Mallick

**Affiliations:** 1 Biomedical Engineering, School of Medicine, Oregon Health and Science University, Portland, OR USA; 2 VA Palo Alto Health Care System, Palo Alto, CA USA; 3 Department of Radiation Medicine, Oregon Health & Science University, 3181 SW Sam Jackson Park Rd, Portland, OR USA; 4 Canary Center at Stanford, Department of Radiology, Stanford University School of Medicine, Stanford, CA USA; 5 Department of Surgery, Stanford University School of Medicine, Stanford, CA USA; 6 Departments of Urology and Medicine, Stanford University School of Medicine, Stanford, CA USA

## Abstract

Platelets, components of hemostasis, when present in excess (>400 K/μL, thrombocytosis) have also been associated with worse outcomes in lung, ovarian, breast, renal, and colorectal cancer patients. Associations between thrombocytosis and cancer outcomes have been made mostly from single-time-point studies, often at the time of diagnosis. Using laboratory data from the Department of Veterans Affairs (VA), we examined the potential benefits of using longitudinal platelet counts in improving patient prognosis predictions. Ten features (summary statistics and engineered features) were derived to describe the platelet counts of 10,000+ VA lung, prostate, and colon cancer patients and incorporated into an age-adjusted LASSO regression analysis to determine feature importance, and predict overall or relapse-free survival, which was compared to the previously used approach of monitoring for thrombocytosis near diagnosis (Postdiag AG400 model). Temporal features describing acute platelet count increases/decreases were found to be important in cancer survival and relapse-survival that helped stratify good and bad outcomes of cancer patient groups. Predictions of overall and relapse-free survival were improved by up to 30% compared to the Postdiag AG400 model. Our study indicates the association of temporally derived platelet count features with a patients’ prognosis predictions.

## Introduction

Platelets are small anucleate peripheral blood cells generated from megakaryocytes in the bone marrow. The primary function of platelets is to stem blood loss after vascular injury. Yet there is increasing evidence that platelet contribute to the pathophysiology of cancer. For instance, several lines of evidence suggest that an interaction between platelets, tumor cells, and endothelial cells enables successful metastasis and worsens the prognosis of cancer patients. More specifically, platelets may play roles in guarding tumor cells from immune elimination and promoting arrest and extravasation of tumor cells^1–3^. In a symbiotic manner, there are also experimental observations suggesting that thrombocytosis is driven by tumor-derived growth factors^4^. Prior studies have shown utility in pre-treatment platelet counts being associated with progression-free and overall survival^5–7^. Thrombocytosis, defined as a platelet count of >400 K/µL of blood, has been observed in cancer patients and it associates with poor prognosis in colorectal^8–11^, breast^12^, lung^13–17^, renal^18–20^, cervical^21^, pancreatic^22^, brain^23^ and ovarian cancers^24,25^ with a 15–40% occurrence.

These prior studies have relied on the binary determination of whether or not platelet counts are abnormal only after a cancer diagnosis has been made, which may be months to years after a malignant transformation has occurred. In addition, prior studies have had limited patient numbers. The most extensive study included hundreds of patients. Here we use a cohort of thousands of cancer patients and leverage clinical data that includes multiple platelet counts for each patient over a four-year period prior to their diagnosis in addition to platelet counts three months following their initial treatment. Thus, the goal of this research is to examine the prognostic power of platelet count-based predictions by incorporating broader temporal measurements. We hypothesize that using platelet counts prior to a cancer diagnosis and after a cancer treatment have added prognostic capabilities as compared to single time-point evaluations of thrombocytosis after a cancer diagnosis is established. Temporal relationships of platelet count and platelet count variability were determined and analyzed with patient outcomes when controlling for stage and cancer site of origin. By looking up to four years prior to diagnosis, we are able to examine each patient’s platelet count variability and how time-of-sampling impact platelet counts and prognosis prediction.

We examined the relationship between platelet counts and patient prognoses for ten features (summary statistics and engineered features) across broad time windows. This study was enabled by access to a veteran lung cancer patient cohort, in which platelet count information was available many years prior to diagnosis. Statistical and machine learning approaches were used to identify which metrics associated with platelet count measurements were associated with relapse-free survival and overall survival in stage-stratified patients. We then adapted this analysis to additional colon and prostate cancer patient cohorts to determine the generalizability of this approach.

## Methods

### Selection and exclusion criteria of veteran patients with lung cancer

The study was conducted retrospectively with the Veteran Affairs’ electronic health record in the Corporate Data Warehouse and Cancer Registry. Compliance with ethical standards was followed. The Stanford University Institutional Review Board deemed the project to be exempt from human subject review. Within these health records, we identified 223,144 lung cancer patients that had been diagnosed between the years 2000 and 2015 and had undergone a therapeutic intervention (surgery, radiation, chemotherapy, immunotherapy or a combination of these). From this, we removed patients with chronic pre-existing conditions associated with essential and reactive thrombocytosis which included chronic myelogenous leukemia, polycythemia vera, myelofibrosis, tuberculosis, hemolytic anemia, rheumatoid arthritis, celiac disease, chronic kidney failure, and diabetes^26^. We then included only records which contained at least yearly platelet counts four years prior to the date of diagnosis and platelet counts recorded between the diagnosis and first treatment, and three months after their first treatment. We identified 21,919 patients in our initial cohort that met these constraints and were included in our analysis. The American Joint Committee on Cancer (AJCC) stage system was used to describe the respective progression of each of the lung cancer patients at the time of diagnosis. Early stage patients are defined as having AJCC “I” or “II” and late stage patients are defined as having AJCC “III” or “IV”. Associations of mean platelet counts within demographic groups (age, gender, cancer stage, race, smoking, treatment received) were compared with either an unpaired t-test or ANOVA. Significance was considered with a p-value less than 0.05.

### Platelet counts, data processing and feature extraction

**Figure 1:**
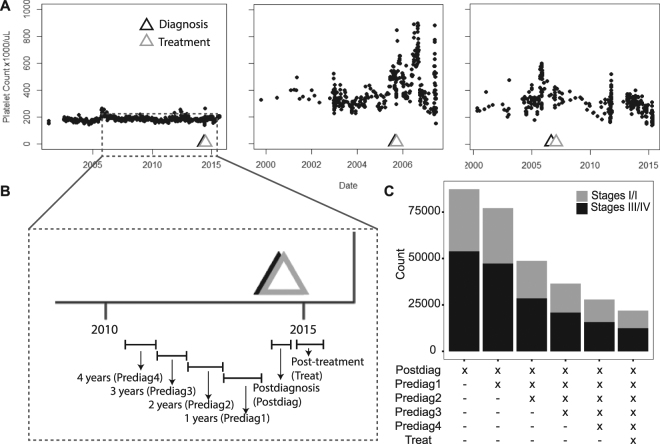
(**A**) Example temporal platelet count information of three patients with annotation of the date of diagnosis and primary treatment. (**B**) Each of the patient’s lab count information is discretized into the following time periods which include Prediag4, Prediag3, Prediag2, Prediag1, Postdiag and Treat, which are respectively fourth, third, second and first year time periods prior to the date of diagnosis, the time interval between the date of diagnosis and the date of the first intervention, and a three-month period following the intervention. (**C**) The size of the study population after incorporating more time periods for early and late stage lung cancer patients.

Platelet counts for all of the patients were queried using Logical Observation Identifiers Names and Codes (LOINC) matching 26515-7, 777-3, 778-1, 13056-7, or 26516-5. Platelet count histories of three example individuals demonstrate the variation in platelet count over time (Fig. 1A). The patient lab count history was parsed into the following time intervals: Postdiagnosis (Postdiag) (between diagnosis and an intervention), Post-treatment (Treat) (three-month period following initial intervention), and the first (Prediag1), second (Prediag2), third (Prediag3), and fourth (Prediag4) yearlong intervals prior to the cancer diagnosis (Fig. 1B). Population size of complete data sets varied as a function of the number of time periods included (Fig. 1C).

Additional measures were also taken to minimize the impact of acute interventions or diseases not covered by the exclusion criteria on platelet count measurements. When multiple platelet counts were measured within a 48-h period (time determined from consult with local physicians), the mean of the counts was obtained to minimize the impact of acute events on platelet count measurements. Distributions of all of the pooled patients’ time between platelet counts (Suppl. Figure 1A,B**)** and the average time between platelet counts on an individual patient basis (Suppl. Figure 1C,D) were significantly altered after applying the 48-h filter. This would suggest that a portion of this population had period of close platelet count monitoring in their health records.

A list of engineered features was derived to describe each of the intervals. Their descriptions are given in Suppl. Table 1. Acute episodes of increases in platelet count were described by features such as peaks, which were determined by an algorithm (http://www.billauer.co.il/peakdet.html) that searched for maximum or minimum within the 75^th^ quantile of all of the platelet counts and classified platelet counts 10 days prior and after the maximum or minimum, time between the peaks (Peaktime), and fraction of platelet counts within a peak (Fraction) (Suppl. Figure 1B) (note: the number of peaks were quantified prior to applying the 48 h filter). Descriptors of platelet counts outside of the normal range included indicators of platelet counts greater than 400 K/μL (AG400, aka thrombocytosis) or 800 K/μL (AG800), the number of the times the platelets exceeded 400 K/μL (Freq). The peak portions of the patients’ platelet count record were masked and summary statistics of baseline platelet counts in each time period were described by the mean, minimum (Min), variance, and maximum (Max), and slope (Suppl. Figure 2A**)**. For patients with only one count in a time period, variances and slope were assumed to be zero.

Each of the engineered features that had been calculated for each patient were averaged on a stage basis for each of the time periods. In order to visualize changes in all of the features over time for each the early and late stage groups, all of the features were scaled to a zero mean and unit variance population (zero median and unit interquartile range for non-normal features). All the derived values were plotted in a heatmap with the features listed as rows and the time periods as column. Each of the squares were color coded to represent positive (red) and negative (blue) deviations from the mean/median.

### Feature Variation Across Surviving and Non-Surviving Populations

To determine the prognostic significance of features for survival and relapse-free survival, the patients were analyzed by whether they did or did not survive/relapse within five years. A Wilcoxon Rank-Sum test was conducted across a subset of the features (AG800, AG400, Freq, Max, Min, Peak, Fraction) within the two groups. The features with less than a 5% false discovery rate across the patient groups were considered to be significant, as determined by the Benjamini-Hochberg procedure.

### Prognosis Prediction and Feature Inference in LASSO Model

All of the features were incorporated in a multivariate logistic regression model with LASSO (least absolute shrinkage and selection operator) regression analysis. The data was separated into training (2/3 of data) and test (1/3 of data) sets. After training and developing the model with the training set, predictions were made on a test set, classifying the patients into two groups of those predicted to survive or not, or recur or not recur, within the five-year time period. Time to death and time to recurrence were determined based on the time between the date of diagnosis and date of death, and the date of diagnosis and date of recurrence, respectively. The LASSO tool was used to reduce the dimensionality of the platelet count features, and then remaining features (only prediagnosis and postdiagnosis data) were put into a logistic regression analysis which investigated 5-year overall survival and recurrence free survival in order to obtain inference on the features. The logistic regression analysis was adjusted for age, race, gender, and tobacco usage. Resulting beta coefficients with p-values <0.05 were considered to be significant and their odds ratios (OR) were tabulated. Kaplan-Meier curves were generated for two patient groups stratified based on being a positive match for any of the significant features with OR > 1.25 or OR < 0.75. Positive matches were defined as a patient being in the top 10% (OR > 1.25) or bottom 10% (OR < 0.75) for a continuous feature, and “True” if a binary variable. The remainder of the patients not matching the criteria formed the second group. The differential significance between the survival curves of the two groups was evaluated by a Log-rank test. The performance of the prediction models (with and without treatment) was measured by generating a receiver operating curve and calculating an area under the curve (AUC) statistic. Additionally, a sensitivity analysis was conducted to isolate the effect of including each prediagnosis interval on prognosis predictions (Suppl. Methods). All statistical analyses were performed using R (Version 1.0.136) and built-in R Packages (dplyr, survminer, survival, ROCR).

### Prognosis Predictions and Inference in Postdiag AG400 Model

Prognosis predictions of patients utilizing the Postdiag AG400 model were also conducted in an effort to emulate previous single time-point measurement studies which measured thrombocytosis around the time of diagnosis. A platelet count was randomly selected from the Postdiagnosis period and was considered to have a “True” AG400 value if it exceeded 400 K/μL. Kaplan-Meier curves were also made in which patients were stratified based on the AG400 variable. The significance of the Kaplan-Meier survival analysis was evaluated by a Log-rank test. Inference on the AG400 variable was done in a logistic regression adjusted by age, gender, race and tobacco usage. The OR were calculated for the Postdiag AG400. Lastly, the performance of using only the Postdiag AG400 was measured with an AUC, and then compared to the longitudinal model’s AUC.

### Comparison of Prognosis Predictions in Patients with Prostate and Colon Cancers

Patients with prostate and colon cancer were evaluated. Using similar search criteria for lung cancers, we identified 271,439 and 57,839 patients with a diagnosis of prostate or colon cancer, respectively. After controlling for pre-existing conditions known to affect platelet counts, and including only records with adequate platelet count records, we included 16,529 prostate cancer patients and 6,050 colon cancer patients, respectively. Similarly, to the lung cancer patient cohort, a Wilcoxon Rank-Sum test was performed between patients that did or did not survive/relapse within 5 years and significant variables were determined with a 5% false discovery rate. The LASSO model and Postdiag AG400 models were again used for feature inference, Kaplan-Meier survival analysis, and the performance of the two models was compared by an AUC.

### Associated Comorbidities and Procedures

Associated procedures and diagnoses were pulled within 30 days of the occurrence of the peaks on an individual basis in 10,000 of the lung cancer patients. The International Classification of Diseases (ICD) codes were made less granular by only using the first three digits and pooled together, with each code only being counted once per patient even if the diagnosis occurred more than once.

### Data Availability

The datasets analyzed during the current study are not publicly available due to veteran patient privacy protection, but de-identified datasets are potentially available from the corresponding author on reasonable request.

## Results

### Lung Cancer Cohort Description

Records from 21,919 patients with a diagnosis of lung cancer were evaluated (Table 1**)**. Of this cohort, the majority of the patients were older than 60 years old (87%) and predominantly male (98%). 9,458 (43%) of the patients were considered to be stage I/II and 12,461 (57%) of the patients that were considered to be stage III/IV at the time of diagnosis. The race breakdown of the cohort was mostly white (82%), but also black (17%), Asian (0.03%) or another race (1.4%). Few patients in the cohort never smoked (2.5%), while 39% previously smoked, 46% were current smokers, and 13% of the patients had unknown tobacco usage. The patients were mostly treated with surgery (29%), chemotherapy (52%), and radiation (44%) and a minority of patients received immunotherapy (0.08%). Mean platelet counts were found to significantly vary among age groups (p < 0.0001), gender (p < 0.0001), cancer stage (p < 0.0001), race (p < 0.0001), smoking (p < 0.0001) and treatment (p = 0.02). The number of 5-year overall survivors from the date of the diagnosis was 6,693 (31%). The number of patients with relapse within 5 years was 1,476 (10%). Five-year OS was found to vary most significantly (>10%) among the cancer stage, smoking history, gender, and treatment groups. (Table 1). The cohort had a median of 17 platelet counts (25^th^–75^th^ percentile = 13–123) prior to their first treatment, which did not significantly vary among the demographic groups.

**Table 1:** Summary Statistics of the Lung Cancer Patients.

Characteristics	Total n (% of 21,919)	# of plt counts median (25^th^–75^th^ percentile)	5-Year OS n (% of Total Column)	Mean Platelet Count (±SD)	p-value (from mean platelet count)
Overall	21,919 (100%)	17 (13–23)	6,693 (31%)	242 ± 86	
Age(years)					
<60	2,819 (13%)	18 (14–24)	1,018 (36%)	259 ± 75	P < 0.0001
≥60	19,100 (87%)	17 (13–23)	5,675 (30%)	240 ± 72	
Gender					
Female	526 (2%)	17 (13–22)	208 (40%)	272 ± 80	P < 0.0001
Male	21,393 (98%)	17 (13–23)	6,485 (30%)	241 ± 72	
Cancer stage					
I/II	9,458 (43%)	17 (13–23)	4,632 (49%)	236 ± 70	P < 0.0001
III/IV	12,461 (57%)	17 (13–23)	2,061 (17%)	247 ± 73	
Race					
Black	3,652 (17%)	18 (14–24)	1,197 (33%)	249 ± 75	P < 0.0001
White	17,875 (82%)	17 (13–23)	5,370 (30%)	241 ± 71	
Asian	76 (0.03%)	16 (13–22)	28 (37%)	252 ± 72	
Other	316 (1.4%)	17 (12–23)	98 (31%)	247 ± 84	
Smoking					
Never	545 (2.5%)	18 (13–24)	215 (40%)	227 ± 60	P < 0.0001
Previously	8,451 (39%)	17 (13–23)	2,560 (30%)	236 ± 70	
Current	10,041 (46%)	17 (13–23)	2,996 (30%)	247 ± 74	
Unknown	2,882 (13%)	18 (14–23)	922 (32%)	242 ± 76	
Treatment*					
Surgery	6,332 (29%)	16 (12–22)	3,580 (57%)	240 ± 69	P = 0.02
Chemotherapy	11,399 (52%)	18 (14–23)	2,584 (23%)	244 ± 70	
Radiation	9,742 (44%)	18 (14–24)	2,466 (25%)	242 ± 72	
Immunotherapy	188 (0.08%)	18 (14–25)	81 (43%)	243 ± 76	
Unknown	1,995 (9%)	17 (13–24)	366 (18%)	242 ± 76	

^*^The total does not add up to 100% because some patients had more than one treatment.

### Time-Dependent Variability of Descriptive Features of Platelet Counts

**Figure 2:**
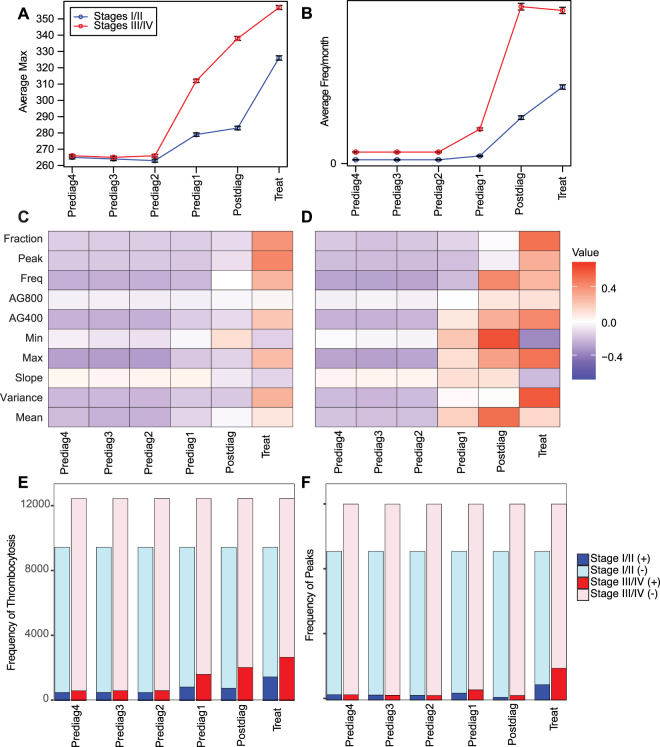
The temporal variations of the features are shown. Averages and standard error of the mean of all of the patients are shown for each of the time periods by stage (Stages I/II – blue, stages III/IV –red) for two example features: (**A**) Max and (**B**) Freq. All of the normalized features are shown over time (**C**,**D**). The average of each feature for each time interval were centered by calculating a z-score from the overall average or median (non-normal data) and standard deviation of all the time periods for (**C**) Stages I/II and (**D**) Stages III/IV patients. Red and blue colors are indicative of features that respectively deviate positively and negatively from an overall average (or median). The quantity of patients with at least one (**E**) thrombocytosis event or (**F**) peak occurrence increases at each of the designated time periods.

We sought to determine how the magnitude of the descriptive features of platelet counts varies over time. As shown in Fig. 2A,B, two example features, Max and Freq, did not change substantially in the Prediag2, Prediag3, or Prediag4. The average Max of the patients did increase in the Prediag1, Postdiag, and Treat intervals, especially in the late stage cancer patients compared to the early stage patients. The average Freq of the early stage lung cancer patients also increased in the Prediag1, Postdiag, and Treat intervals, but in the late stage patients, there was a slight decrease in Freq in the Treat phase (Fig. 2A,B). We also plotted a scaled heatmap of feature variation for early and late stage cancer patients, respectively (Fig. 2C,D). The changes in the variables over time could be described in three discernable patterns: (1) Variables that did not change over any of the time periods, (2) variables that did not change in Prediag4, Prediag3, or Prediag2 but then experienced relative increases going from Prediag1to Postdiag to Treat intervals, and (3) variables that did not change in Prediag4, Prediag3, or Prediag2 and then increased in the Prediag1 and Post periods and then decreased in the Treat period. The variable that followed pattern 1 was *Early Stage*: AG800. The variables following pattern 2 include: *Early Stage*: Fraction, Peak, Freq, AG400, Max, Variance, Mean and *Late Stage:* Fraction, Peak, Freq, AG800, AG400, Max, Variance. The variables following pattern 3 include: *Early Stage:* Min and Slope and *Late Stage*: Freq, Min, Slope, and Mean. Lastly, in addition to measuring the overall means of each of the variables, we also enumerated the frequency of patients that had at least one peak or thrombocytosis event in each of the time periods of interest. The percentage of patients that had at least one peak ranged between ~2%–10% for early stage patients and ~2% to 16%, with the higher end of the range occurring in the Treat period. The percentage of patients experiencing thrombocytosis events was ~5%–16% for early stage patients and ~5% to 22% for late stage patients with the upper end of the ranges occurring in the Prediag1, Postdiag, and Treat periods (Fig. 2E,F). Overall, we demonstrate that there was little change in the platelet variables in the Prediag4, Prediag3, and Prediag2 periods and much more variability in the Prediag1, Postdiag, and Treat phases, particularly in the late stage patients. Additionally, a decrease in the Treat variable for Freq, Min, Slope, and Mean could be indicative of the effects of the treatments.

### Feature Variation Across Surviving and Non-Surviving Populations

**Figure 3:**
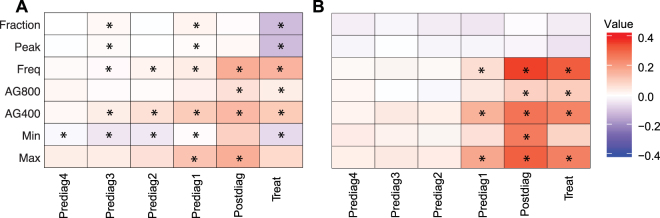
The differences between the normalized population that survived less than five years versus the patients that survived more than five years in (**A**) early stage and (**B**) late stage cancer patients. Red and blue colors are indicative of features that respectively have an increased or decreased signal in the population that survived less than five years compared to the population that survived more than five years. Each of the features within the two population groups were compared using a Wilcoxon Rank-Sum test. Each * is indicative of tests with P-values regarded as having less than a 5% false discovery rate.

A subset of the features was compared between the populations that did or did not survive/recur within a five-year period by using a Wilcoxon Rank-Sum test for early and late stage cancer patients to determine which variables significantly varied across the population groups. The normalized differences between the population that survived or not are shown for early (Fig. 3A) and late (Fig. 3B) stage cancer patients and their respective P-values and adjusted P-values are given in Suppl. Table 2. Overall, there were significant differences found between lung cancer patients that did not survive versus those that did in early stage lung cancer patients in Fraction, Peak, Freq, AG800, AG400, Min, Max, with significance in each spanning between 2–5 time periods. Freq, AG800, AG400, and Max were elevated, and Min was decreased in populations that did not survive. Fraction and Peak trends depended on the time period; they were increased during the Prediag3 and Postdiag1 phases and then decreased in in the Treat phase in non-surviving populations (Fig. 3A). Differences in late stage cancer patients between non-surviving and surviving populations occurred in Freq, AG800, AG400, Min and Max, with each spanning 1–3 time periods (all in Prediag1, Postdiag, and Treat periods). All of the variables were elevated in the population that did not survive (Fig. 3B**)**. The adjusted p-values for the early stage cancer patients ranged from 3 × 10^−7^–5 × 10^−2^ for AG400, AG800, Fraction, Peak, Freq, Min and Max (Suppl. Table 2), while in the late stage cancer population, the adjusted p-values ranged from 2 × 10^−13^–5 × 10^−2^ for Freq, AG800, AG400, Min and Max (Suppl. Table 2). For early stage relapse-free survival, only Postdiag AG800 (adj. p-values: 3 × 10^−2^) was found to be significantly different between populations with relapse versus those without (Suppl. Table 2). There is significant feature variation across surviving and non-surviving populations with temporal variation. Early stage patients had significant variables in all of the time periods, whereas the late stage patients were limited to Prediag1, Postdiag, and Treat periods.

### Feature Inference from the adjusted LASSO and Postdiag AG400 Model

**Figure 4:**
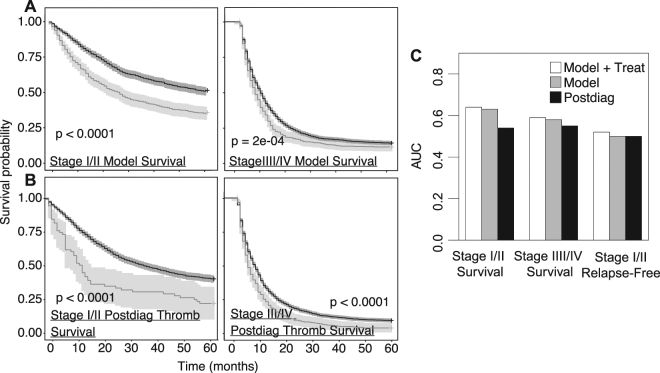
Kaplan Meier curves for lung cancer patient survival were either based on **(A**) LASSO model-derived features with OR > 1.25 or OR < 0.75 in adjusted logistic regression. Positive matches were defined as a patient being in the top 10% (OR > 1.25) or bottom 10% (OR < 0.75) for a continuous feature, and “True” if a binary variable (**B**) or a True Postdiag AG400. Positive matches are represented in light grey, and negative matches are represented in dark grey. (**C**) Overall description of the performance of the prediction methods with an AUC metric. The white bars indicate the AUCs obtained in the LASSO Model with the inclusion of the post-treatment data, the grey bar indicates the AUCS obtained with just the LASSO Model, and the black bars indicate the AUCs obtained from just using the Postdiag AG400 model.

The platelet features obtained in the LASSO model or the Postdiag AG400 variable were incorporated into an adjusted logistic regression model with 5-year survival/relapse-free survival outcomes. The logistic regression models were adjusted for age, gender, race, and tobacco usage. In the LASSO model, it was determined that temporal platelet count features were independently associated with survival and relapse-free survival (Suppl. Table 3). For example, in the case of survival outcomes of stage I/II lung cancer patients, Postdiag Max, Prediag1 Max, Prediag1 Min, Prediag2 AG400, and Prediag3 AG400 had ORs of 1.4 (95^th^ CI 1.2–1.6), 1.3 (95^th^ CI 1.2–1.5), 0.73 (95^th^ CI 0.65–0.81), 2.5 (95^th^ CI 1.6–3.8), and 1.6 (95^th^ CI 1.1–2.4), respectively (only listing OR > 1.25 or < 0.75). In an adjusted logistic regression model for stage I/II lung cancer patient survival with the Postdiag AG400 variables, Postdiag AG400 had an OR of 1.6 (95^th^ CI 1.3–1.9) (Suppl. Table 4). No platelet variables were found to be significant with 5-year relapse-free survival. Kaplan Meier curves for lung cancer patient survival were generated by stratifying the patients either based on (1) LASSO model-derived platelet count features with OR > 1.25 or OR < 0.75 in an adjusted logistic regression model, or (2) the Postdiag AG400 variable. The patient groups for both models were plotted in a Kaplan Meier curve for early and late stage survival (Fig. 4A,B). Statistical significance was found between the two groups in all of the scenarios, even for small effect sizes or largely variable groups (found in all the Postdiag AG400 models) due to the large number of patients in the study.

### Relapse-Free Survival and Overall Survival Prediction as a Function Amount of Time Incorporated in the Model

A sensitivity analysis was conducted (Suppl. Methods) to determine the minimum amount of time intervals of platelet count information to improve relapse-free survival and overall survival predictions in patients with lung cancer. Each of the time periods was successively introduced into a random forest model and assessed with an AUC measurement. In stage I/II patient 5-year survival, the AUC increased the most from 0.53 to 0.59 after the inclusion of the Prediag1 data. Adding Prediag2, Prediag3, and Prediag4 only added marginal benefit and lead to a slight increase of AUC to ~0.6. In stage III/IV 5-year survival, AUCs with just Postdiag and Prediag1 and Postdiag together were ~0.56. Adding Prediag2, Prediag3, Prediag4 actually slightly reduced the performance of the model to an AUC ~0.54. In summary, it was found that incorporation of more time periods prior to diagnosis is the most beneficial for stage I/II survival of patients with lung cancer, whereas only a one-year history prior to diagnosis was required for late stage survival prediction, and no additional benefit was attained by incorporating more platelet count history for all recurrence predictions (Suppl. Figure 3).

### Comparison of LASSO Model to Postdiag AG400 Model

The performance of the LASSO model with and without the treatment data and Postdiag AG400 model were compared via AUCs. AUCs for early and late stage survival, and relapse-free survival were 0.62, 0.58, and 0.50. If the treatment data was added to the LASSO model, some improvement was found, increasing the resulting AUCs to be respectively be 0.63, 0.59, and 0.54. The AUCs from the Postdiag AG400 Model were 0.52, 0.52, and 0.50 from (Fig. 4C). In summary, it was found that incorporating the treatment and the prediagnosis data lead to improved predictions compared to just assessing thrombocytosis between the diagnosis and first treatment.

### Prognosis Predictions Across Other Cancers

**Figure 5:**
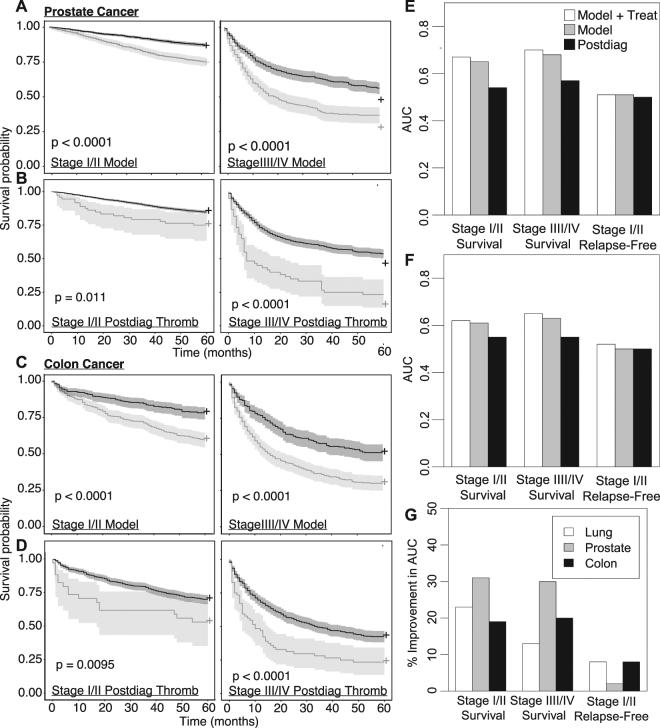
Kaplan Meier curves for prostate and colon cancer patient survival were either based on (**A**,**C**) LASSO model-derived features with OR > 1.25 or OR < 0.75 in an adjusted logistic regression model. Positive matches were defined as a patient being in the top 10% (OR > 1.25) or bottom 10% (OR < 0.75) for a continuous feature, and “True” if a binary variable or (**B**,**D**) a True Postdiag AG400. Positive matches are represented in light grey, and negative matches are represented in dark grey. Overall description of the performance of the two prediction methods with an AUC metric with (**E**) prostate and (**F**) colon cancer patients. The white bars indicate the AUCs obtained in the LASSO Model with the inclusion of the Treat data, the grey bar indicates the AUCS obtained with just the LASSO Model, and the black bars indicate the AUCs obtained from just using the Postdiag AG400 model. (**G**) An overall summary of how the prognosis prediction of early and late survival, and early stage relapse improve by using the LASSO model + Treat instead of the Postdiag AG400 model. These improvements were calculated based on the AUCs obtained with the platelet count features only.

To investigate the generality of temporal platelet count analysis, we repeated our analysis for cohorts of patients with prostate (Suppl. Figure 4A–B) or colon cancer (Suppl. Figure 5C–D) (summary statistics given in (Suppl. Tables 5 and 6)). The number of 5-year overall survivors from the date of the diagnosis was 12,510 (75%) and 3,527 (58%) in prostate and colon cancer patients, respectively. The number of patients with relapse within 5 years was 435 (3%) and 163 (5%), respectively, in prostate and colon cancer patients. Patients were stratified into groups that survived for five years versus those that did not. The normalized differences between the two populations are shown for early and late stage overall survival of prostate (Suppl. Figure 5A–B) and colon cancer patients (Suppl. Figure 5C–D) and their respective P-values and adjusted P-values are given in Suppl. Tables 7–8. The OR of the significant features were calculated for the age-adjusted LASSO and Postdiag AG400 models (Suppl. Tables 9–11**)**. Briefly, similarly to lung cancer patients, several platelet count features were found to be significant in outcomes of stage I/II and stage III/IV survival, but no platelet variables were found to be significant with 5-year relapse-free survival as the outcome. Stratification of patients occurred in the same manner as the lung cancer patients for both of the models. Kaplan Meier curves of the patient groups are demonstrated for early and late stage survival (Fig. 5A,C) in prostate and colon cancer respectively, using the LASSO model, in comparison to the Postdiag AG400 model (Fig. 5B,D) (prostate and colon cancer respectively). In prostate cancer, AUCs for early and late stage survival, and relapse-free survival (treatment added to model in AUCs in parentheses) were 0.65 (0.67), 0.68 (0.70), and 0.50 (0.50) for the model and 0.51, 0.54, and 0.50 from only using Postdiag AG400 Model (Fig. 5E). In colon cancer, AUCs for early and late stage survival, and relapse-free survival were 0.61 (0.62), 0.63 (0.65), and 0.50 (0.54) for the model and 0.51, 0.54, and 0.50 from the Postdiag AG400 model (Fig. 5F). The performance of the LASSO model with the treatment data was compared to the Postdiag AG400 model. Accounting for the LASSO model including the treatment data, lung cancer predictions were improved by ~20% for early stage survival, and ~ a 10% improvement was attained for predictions of late stage survival and early stage recurrence. Predictions in prostate cancers were improved by ~30% for early and late stage survival and a minor 2% improvement for early stage relapse-free survival. In the case of colon cancer, predictions improved by ~20% for early and late stage patient survival and a minor improvement was found for early stage relapse-free survival predictions (Fig. 5G).

## Discussion

In this study, we explored platelet count histories in a cohort of veteran patients with lung, prostate, and colon cancer, by examining their platelet count histories in the four years prior to their diagnosis, between their diagnosis and treatment, and three months following their first treatment. A set of features describing each time period was inputted into a binary classification model to predict survival and relapse-free survival. It was demonstrated that longitudinal platelet count features are associated with survival and relapse-free survival in an adjusted model. Prognosis predictions could be improved by ~20–30% by incorporating historic information about platelet counts compared to the Postdiag AG400 model as a function of the type of cancer and stage of the patient.

Many studies have found that perioperative thrombocytosis is associated with worse lung cancer patient outcomes^13,15,16^. For example, Aoe *et al*. found 16% of the patients manifested thrombocytosis at the time of their first evaluation and this resulted in worse patient outcomes (HR: 1.29 95^th^ CI 1.02–1.64)^13^. Yet in another study, no relationship was found between the preoperative thrombocytosis and survival (p = 0.067) in the management of colorectal cancer^27^. Such differences could be due to the studies not accounting for the temporal variation of the platelet count data. Platelet counts are known to vary substantially within a person, even within a few days. We also investigated the prognostic value of postdiagnosis/pretreatment thrombocytosis in order to compare our work to previous studies. The counts exceeding the threshold did carry a worse prognosis (HR: 1.1 95^th^ CI 1.05–1.20), similarly to what the other studies reported, but this method had low sensitivity in recovering the patients with worse prognosis. For example, in early stage lung cancer patients, when we stratified patients based on whether they experienced postdiagnosis thrombocytosis, only ~6% of the patients with less than five-year survival were recovered, whereas our model identified ~68% of the patients with less than five-year survival.

Our model incorporated many engineered features that would describe the behavior of the platelet counts within a given time period. Some of the features were chosen to represent markers of baseline characteristics (Slope, Mean) and other features expressed the acute-like responses in each of the individual’s platelet counts. According to the LASSO model, mainly acute-like features, such as Max, Min, Freq, and Fraction were the strongest contributors to the model prediction. Of interest, it was found was that patients having a low Min in their platelet count histories was just as high risk as having a high Max. In order to learn more about the etiology of the peak events, associated diagnoses and procedures within a month of peaks were queried for each of the cancer patients that had peak events. A variety of illnesses and procedures were recovered, with essential hypertension and transfusions as the most frequent diagnosis and procedure, respectively (Suppl. Figure 6).

It remains unknown whether the raised platelet counts are related to the cancer itself. It has been suggested that tumor-derived thrombopoietic cytokines lead to paraneoplastic thrombocytosis^4^. Some clinical case control studies have suggested that the presence of thrombocytosis is positively associated with cancer^28^. Yet there were no clear indications from our data that the increase in platelet count was aligned with the progression of the cancer – platelet counts increased the most around the time of diagnosis for early and late stage cancer patients. It is more likely that the increase could be related to a malignancy and illness that lead to the detection of the cancer and that the platelet counts are a surrogate measure of health. Though it is worth noting that the features describing platelet counts were higher in magnitude for the late stage cancer patients close to the time of diagnosis as compared to the early stage lung, colon, and prostate cancer patients. What is important is that the presence of elevated platelet counts is associated with a worse prognosis. This may be reflective of a role for platelets in the promotion of the metastatic process. Whether this is mechanistically involved with cancer progression or provides justification for pharmacological inhibition of platelet activity in patients with cancer patients cannot be determined from this study^29–32^.

Interestingly, the performance of the LASSO model was marginally improved with the inclusion of the treatment data. The treatment data carried some additional unique signal that would be missed only looking at the prediagnosis and postdiagnosis data. Notably, according to the Wilcoxon Rank Sum test, variables such as Freq, AG800, AG400, and Max increased in patients that had worse prognosis during the treatment phase, while other variables such as Fraction, Peak, and Min declined. The treatment data might help determine the success of the particular therapy.

In addition to examining temporal platelet count information to make improved predictions in lung cancer patients, we also expanded our analysis to include prostate and colon cancer patients to determine whether this analysis could be used for other cancer subtypes. In our study, we found that using historical platelet counts improved prediction efforts the most in prostate cancer (early and late stage patient survival both by ~30%). In colon cancer, predictions improved by ~20% for early and late stage survival and slightly (8%) for relapse-free survival. Yet in lung cancer, significant improvements were only made for early stage patient survival (23%). One explanation for the differences would be that lung cancer patients overall have worse survival than prostate and colon cancer patients - while lung cancer patients have an overall survival of 31% (49% for stage I/II, 17% for stage III/IV), prostate cancer patients had an overall survival of 77% (82% for stage I/II, 54% for stage III/IV), and colon cancer patients had an overall survival of 58% (44% for stage I/II, 68% for stage III/IV). In the case where a patient’s prognosis is already poor, there might be less sensitivity to using platelet counts as a measure of prognosis. Future studies need to be conducted to delineate whether prediction improvements are a function of the overall prognosis of the patients. It would also be of interest to determine if there is a correlation between cancers that are most likely to metastasize through the bloodstream (more platelet and circulating tumor cell interactions), as opposed to the lymphatic system, have increased improvements in prediction accuracy.

Our primary goal in this study was to determine whether temporal evaluation of platelet counts preceding a cancer diagnosis was superior to a single time-point evaluation, building off prior studies showing an association with thrombocytosis at the time of diagnosis with poorer patient outcomes. Our analysis showed that abnormal platelet counts up to a year prior to the diagnosis of cancer was better able to delineate patients with similar stage cancers and probability for five-year survival. We utilized the VA patient data, which is unique in its inclusion of a nationwide patient record database that includes all components of a veteran’s health record which is not available in cancer specific registries which contain only pre-specified patient characteristics related to their cancer diagnosis and treatment. As such, we provide a proof-of-principle study that demonstrates perturbations in seemingly unrelated patient health information that may hold value in more accurate patient prognostics.

In future studies, we will also explore the temporal variation of immune responses and how they fluctuate compared to platelet responses. Other studies have reported preoperative platelet to lymphocyte and neutrophil to lymphocyte ratios, CRP, and albumin as promising metrics for prognosis predictions^33–38^. A limitation in this study is that we only included patients that had a four-year platelet count history, as we were still able to maintain a population of 10,000+ and could go back far enough to learn which time points are critical in determining a patient’s prognosis. Of course, the demographics of a veteran population with this platelet count history do not represent that of the general population, which is a major limitation of this approach.

Nevertheless, in this study, we leveraged platelet count in a large patient cohort and demonstrated prognostic value in deriving a set of features to describe periods of time prior to a cancer diagnosis. Overall, we delineated temporal platelet count variability and found that predictions leveraging temporal platelet count information, as opposed to just measuring platelet counts around the time of treatment, can improve prognosis predictions in a stage and type of cancer-dependent manner.

## Electronic supplementary material

Supplementary Information
