# Supplementary material for: A Temporal Examination of Platelet Counts as a Predictor of Prognosis in Lung, Prostate, and Colon Cancer Patients
Source: Sci Rep. 2018 Apr 26;8:6564. doi: 10.1038/s41598-018-25019-1 (PMC5920102; doi:10.1038/s41598-018-25019-1)
Supplement: Supplementary file 1 — Supplementary Information [file 41598_2018_25019_MOESM1_ESM.pdf]

A Temporal Examination of Platelet Counts as a Predictor of Prognosis in Lung, Prostate, and Colon Cancer Patients

Joanna L. Sylman[1,2,4], Hunter B. Boyce[4], Annachiara Mitrugno[1], Garth W. Tormoen[3], I-Chun Thomas[6], Todd H. Wagner[2,5], Jennifer S. Lee[2], John T. Leppert[2,6], Owen J. T. McCarty[1], Parag Mallick[4]

[1]Biomedical Engineering, School of Medicine, Oregon Health and Science University, Portland, OR; [2]VA Palo Alto Health Care System, Palo Alto, CA; [3]Department of Radiation Medicine, Oregon Health & Science University, 3181 SW Sam Jackson Park Rd, Portland, OR; [4]Canary Center at Stanford, Department of Radiology, Stanford University School of Medicine, Stanford, CA, [5]Department of Surgery, Stanford University School of Medicine, Stanford, CA, [6]Departments of Urology and Medicine, Stanford University School of Medicine, Stanford, CA

Corresponding author: Joanna L. Sylman
Department of Radiology
Stanford University
3155 Porter Dr., Stanford, CA 94304
Phone: 650-721-3089
Email: jsylman@gmail.com

Keywords:
thrombocytosis
prognosis
platelets
cancer

The authors declare no potential conflicts of interest

SUPPLEMENTAL MATERIALS

SUPPLMENTAL METHODS

Sensitivity Analysis on Time Intervals

The effect of the amount of time intervals included in the prognosis prediction was estimated by conducting a sensitivity analysis with a random forest decision tree. A random forest was the chosen model instead of the LASSO method in order to maintain the feature space dimensionality. The random forest consisted of 200 trees and the number of variables that were randomly sampled at each terminal node was set to be equal to the square root of the number of total variables. The random forest was trained with 2/3 of the data and the resulting model was used to make predictions remaining 1/3 of the data. The outcomes of interest were the overall survival, or relapse-free survival with censoring applied at 5 years for both. In the case of the relapse-free survival, the patients that died before the end of the 5-year time period that did not have a recurrence were censored. Each of the time periods were added into the model sequentially. The optimal time periods to predict 5-year overall survival and relapse-free survival were investigated by a receiver operating curve analysis and comparing the area under the curve (AUC) as a function stage.

SUPPLEMENTAL FIGURES

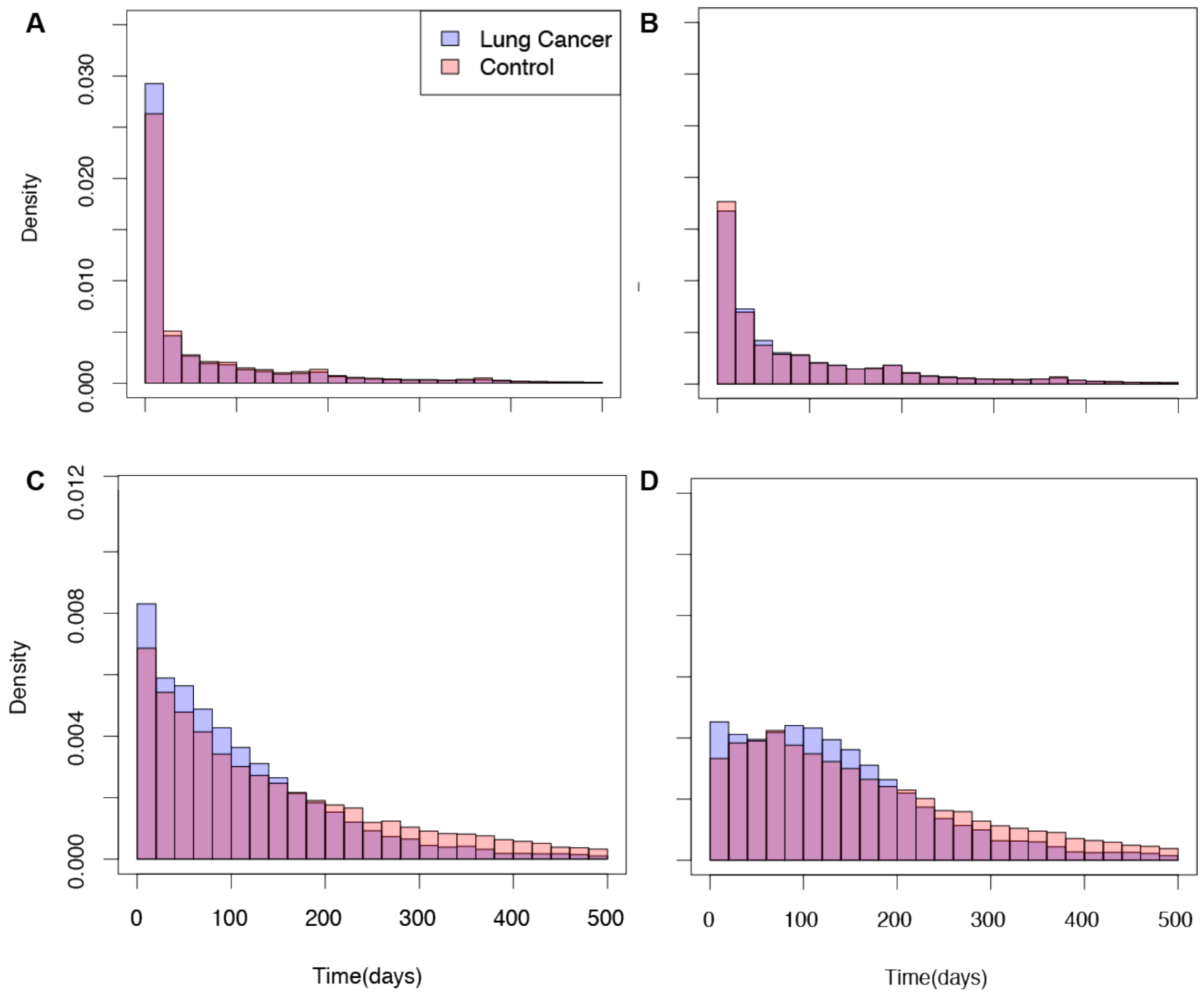

Suppl. Figure 1: The amount of time between platelet counts before and after applying 48 h filtering algorithm in lung cancer patient platelet counts compared to an age-matched control population. The density of all times between platelet counts are pooled together (A) with and (B) without the 48-h filter. The density of the average time between platelet counts on a patient basis are pooled together (C) with and (D) without the 48-h filter.

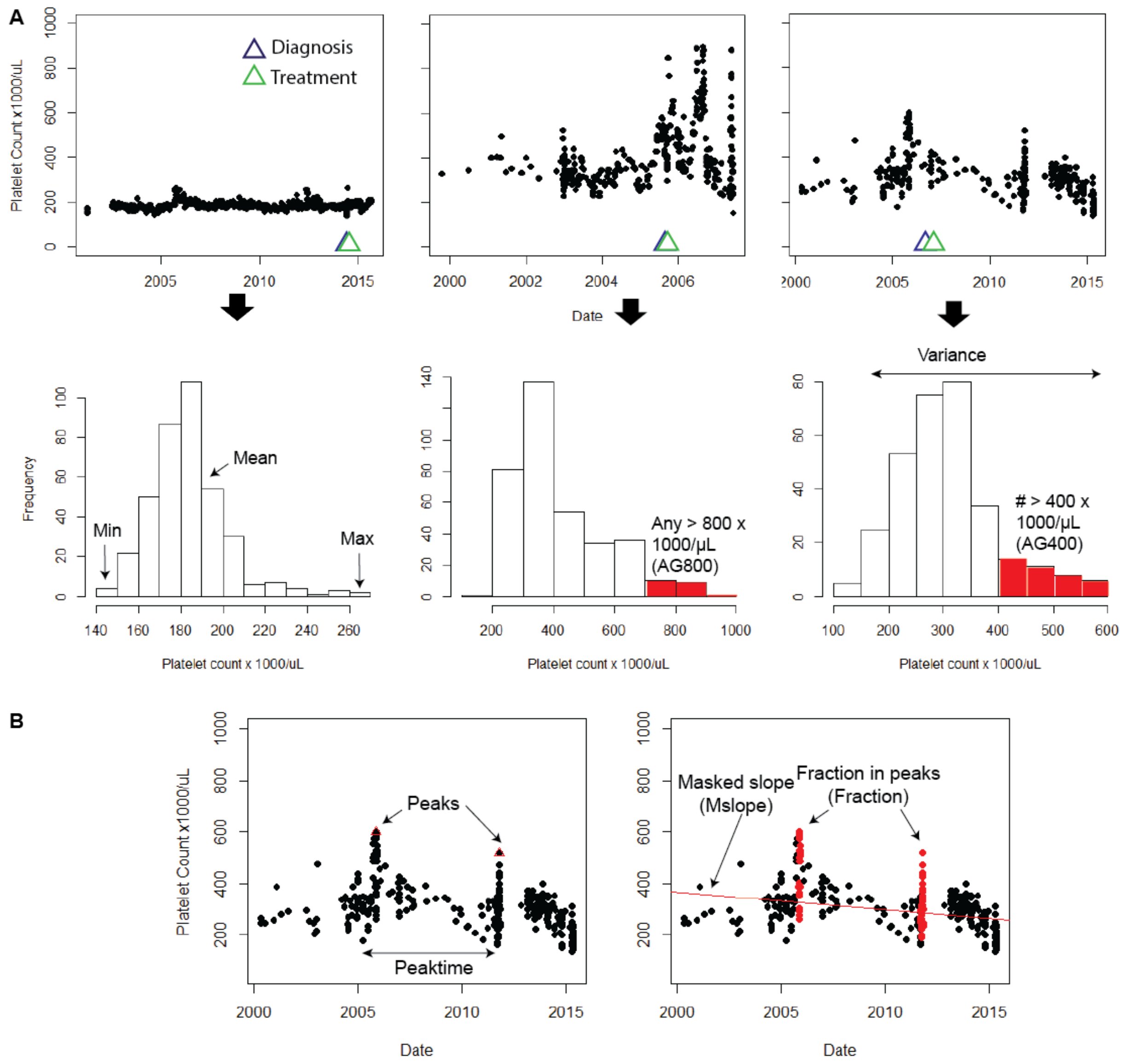

Suppl. Figure 2: Derivation of the features from the lung cancer patient platelet count information. (A) Each of the patient's temporal platelet counts were converted to histograms, in which the variables of interest are demonstrated. (B) Time dependent features can be mined for each of the patients and a peak detection algorithm is applied to determine spikes where patient's platelet counts go outside of their 75th quantile. A masked slope can be determined based off the platelet counts remaining after spikes in platelet counts have been removed.

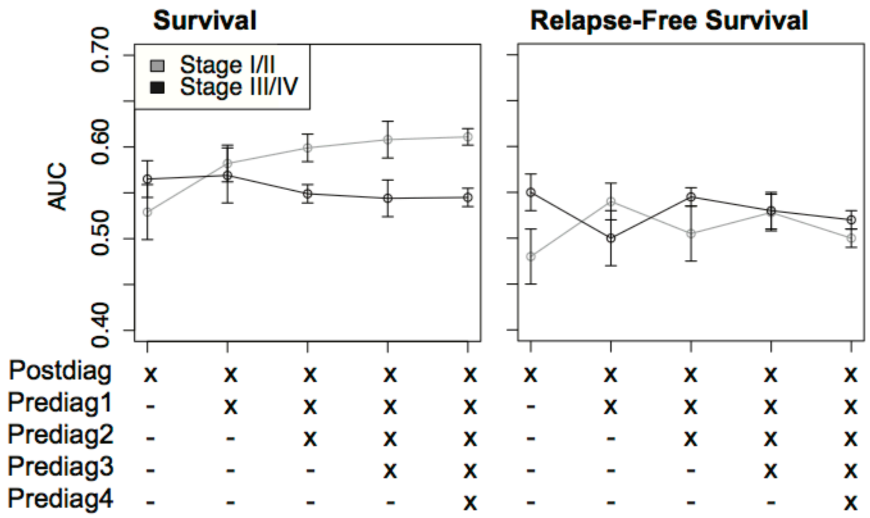

Suppl. Figure 3: All of the parameters were incorporated into a random forest to build a model that was used to predict the overall survival and relapse-free survival of early and late stage cancer patients. The area under the curve (+/- standard error of the mean) obtained from a test set from a trained random forest are shown for each of the time periods for overall survival and relapse-free survival.

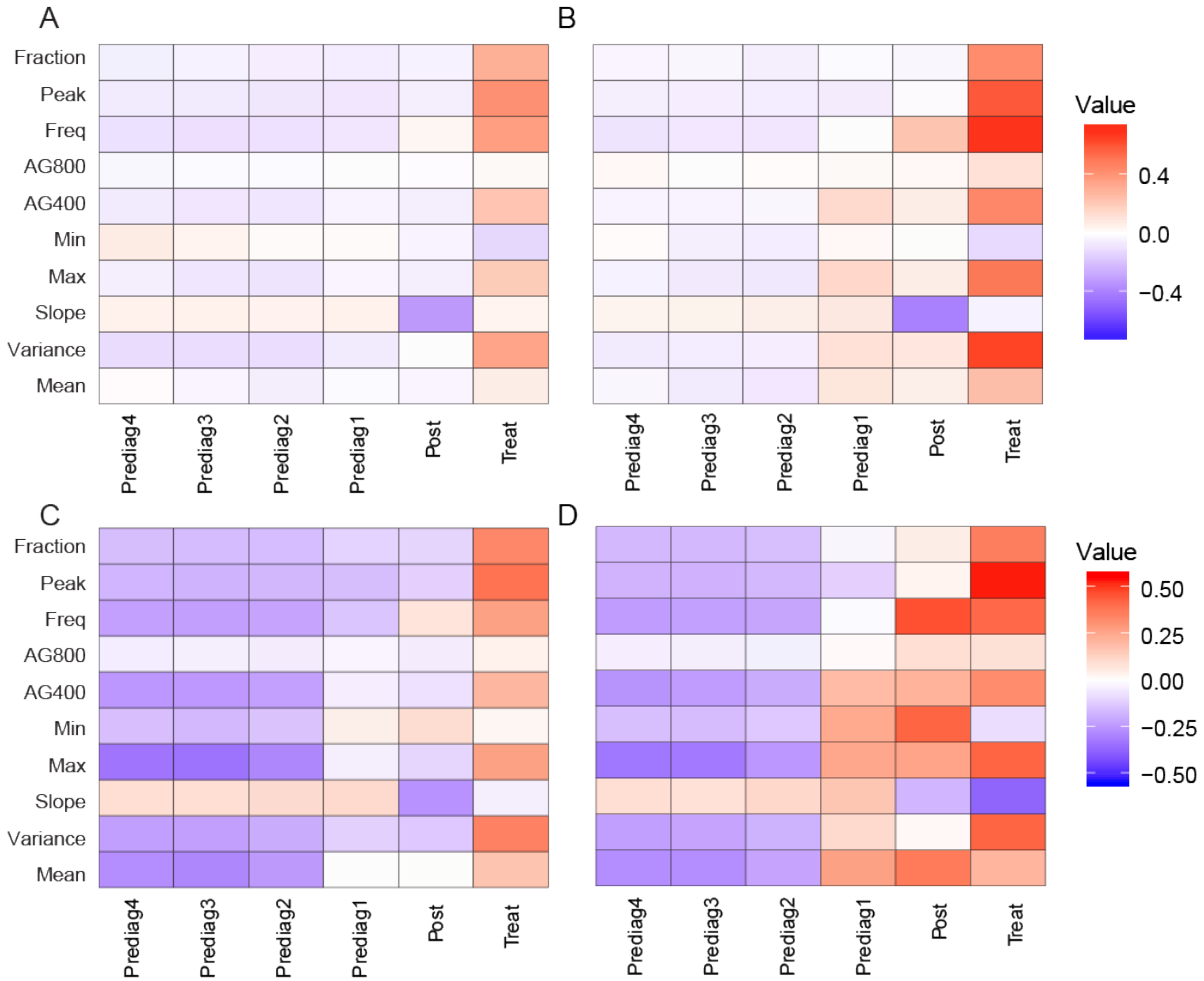

Suppl. Figure 4: The average of each feature for each time interval were centered by calculating a z-score from the overall average (or median for non-normal data) and standard deviation of all the time periods for Stages I/II and Stages III/IV patients in (A-B) prostate and (C-D) colon cancer patients. Red and blue colors are indicative of features that respectively deviate positively and negatively from an overall average.

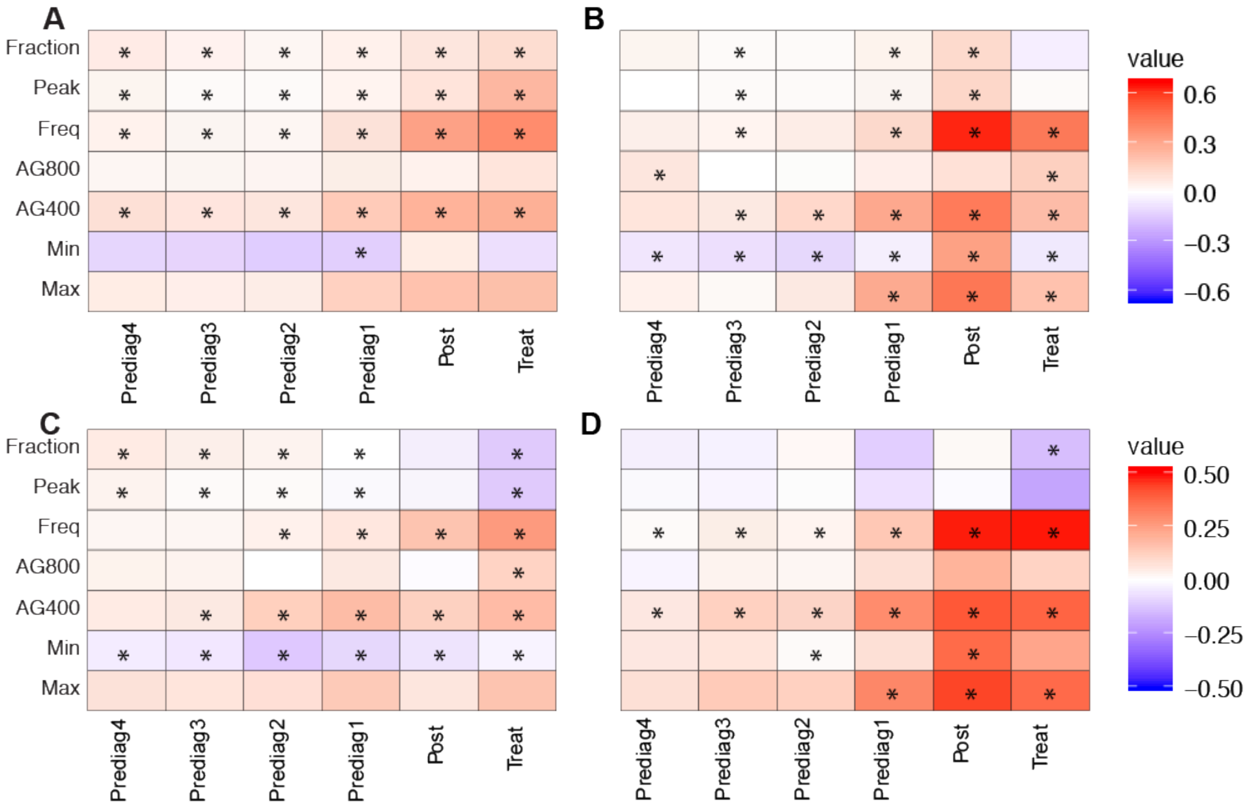

Suppl. Figure 5: The average of each feature for each time interval were centered by calculating a z-score from the overall average (or median for non-normal data) and standard deviation of all the time periods for Stages I/II and Stages III/IV patients in (A-B) prostate and (C-D) colon cancer patients. Red and blue colors are indicative of features that respectively deviate positively and negatively from an overall average.

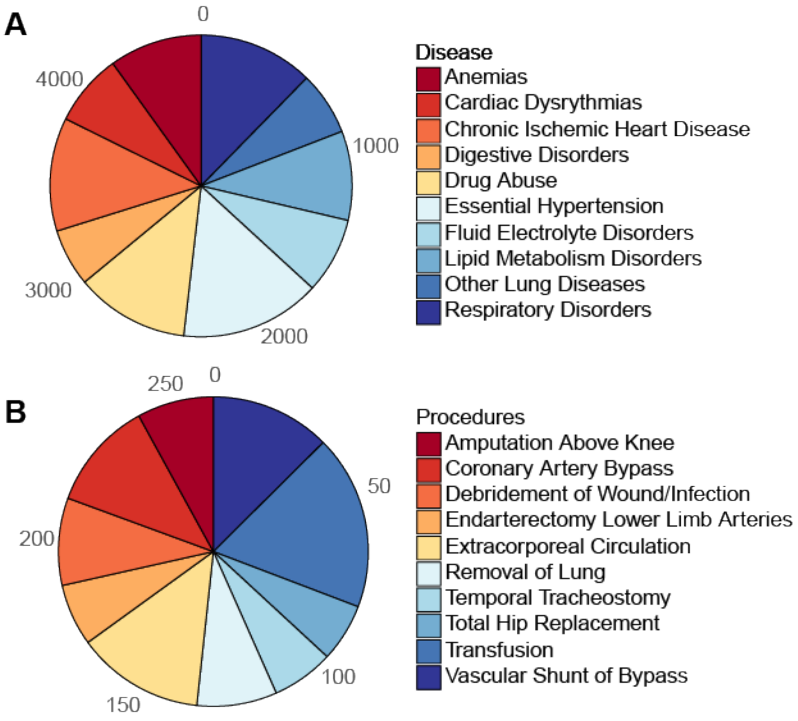

Suppl. Figure 6: Association of peaks with procedures and diseases. The pie charts show the top (A) diseases and (B) procedures that were present in the patients during peak episodes.

SUPPLEMENTAL TABLES

Suppl. Table 1: Derived Feature Descriptions

Features	Feature Description	Type	Units
Variables applicable for every time period			
Mean	Average platelet count	Numeric	K/ µL
Variance	Deviation of platelet counts from the mean	Numeric	$(K/\ \mu L)^2$
Max	Maximum platelet count	Numeric	K/ µL
Min	Minimum platelet count	Numeric	K/ µL
Median	Median platelet count	Numeric	K/ µL
Peak	Number of peaks found in platelet count histories	Integer	None
Fraction	Fraction of platelet counts that are found within a peak	Numeric	Fraction
Peaktime	Average time between peaks in platelet counts	Integer	days
AG400	Any platelet counts > 400K/ µL	Logical	T/F
AG800	Any platelet counts > 800K/ µL	Logical	T/F
Freq	Number of platelet counts > 800K/ µL	Integer	None
Slope	Masked slope (Platelet count × 1000/µL)/month after spike removal	Numeric	(K/ µL)/month
Overall Features			
Stage	The stage of the patient at diagnosis	Categorical	I/II/III/IV
Age	The age of the patient at diagnosis	Numeric	Years
Gender	The gender of the patient	Categorical	F/M

Suppl. Table 2: Wilcoxon Rank Sum Test P-values and Adjusted P-values* in Lung Cancer Patients

		Lung Stage I/II Survival		Lung Stage III/IV Survival		Lung Stage I/II Relapse-Free Survival	
Variables	**Period**	**P-value**	**Adjusted P-value**	**P-value**	**Adjusted P-value**	**P-value**	**Adjusted P-value**
**Max**	Postdiag	9.64E-04	2.95E-03	1.94E-13	3.57E-12	8.13E-01	9.31E-01
	Prediag1	3.40E-04	1.56E-03	1.43E-07	8.76E-07	5.82E-01	8.79E-01
	Prediag2	2.66E-02	5.05E-02	6.49E-02	1.62E-01	6.32E-01	8.79E-01
	Prediag3	5.15E-02	9.15E-02	2.23E-01	3.84E-01	6.36E-01	8.79E-01
	Prediag4	5.17E-02	9.15E-02	7.71E-01	7.85E-01	7.85E-01	9.18E-01
	Treat	7.59E-01	7.73E-01	1.45E-05	6.66E-05	3.22E-01	7.47E-01
**Min**	Postdiag	6.61E-01	7.00E-01	1.18E-12	1.62E-11	7.22E-01	8.79E-01
	Prediag1	2.85E-06	1.96E-05	5.33E-01	5.99E-01	7.35E-01	8.79E-01
	Prediag2	1.53E-04	7.65E-04	8.64E-01	8.64E-01	6.88E-01	8.79E-01
	Prediag3	8.94E-04	2.89E-03	4.96E-01	5.81E-01	6.19E-02	4.21E-01
	Prediag4	2.45E-02	4.81E-02	4.10E-01	5.64E-01	6.19E-01	8.79E-01
	Treat	1.16E-14	3.18E-13	4.94E-01	5.81E-01	2.28E-01	7.47E-01
**AG400**	Postdiag	9.39E-07	8.61E-06	1.75E-08	1.60E-07	6.64E-01	8.79E-01
	Prediag1	4.79E-03	1.10E-02	1.16E-06	5.86E-06	6.16E-01	8.79E-01
	Prediag2	1.99E-08	2.74E-07	8.33E-02	1.91E-01	6.95E-01	8.79E-01
	Prediag3	4.15E-04	1.63E-03	3.24E-02	9.58E-02	3.39E-01	7.47E-01
	Prediag4	1.17E-01	1.75E-01	4.33E-01	5.68E-01	1.29E-01	5.43E-01
	Treat	1.00E-02	2.21E-02	8.13E-08	5.59E-07	9.75E-01	9.85E-01
**AG800**	Postdiag	2.89E-03	7.57E-03	6.20E-03	2.27E-02	9.79E-04	2.69E-02
	Prediag1	2.30E-02	4.69E-02	4.06E-01	5.64E-01	3.05E-01	7.47E-01
	Prediag2	6.63E-02	1.10E-01	5.26E-01	5.99E-01	1.18E-01	5.41E-01
	Prediag3	1.56E-01	2.26E-01	2.91E-01	4.71E-01	2.80E-01	7.47E-01
	Prediag4	1.89E-01	2.66E-01	4.44E-01	5.68E-01	1.68E-02	1.90E-01
	Treat	2.83E-01	3.80E-01	1.43E-02	4.93E-02	4.13E-01	8.44E-01
**Freq**	Postdiag	4.75E-07	5.22E-06	6.57E-09	7.23E-08	5.97E-01	8.79E-01
	Prediag1	3.75E-03	9.36E-03	1.17E-06	5.86E-06	6.83E-01	8.79E-01
	Prediag2	1.99E-08	2.74E-07	8.13E-02	1.91E-01	6.83E-01	8.79E-01
	Prediag3	3.86E-04	1.63E-03	3.34E-02	9.58E-02	3.30E-01	7.47E-01
	Prediag4	1.05E-01	1.66E-01	4.25E-01	5.68E-01	1.38E-01	5.43E-01
	Treat	3.93E-03	9.40E-03	2.63E-08	2.07E-07	9.67E-01	9.85E-01
**Peak**	Postdiag	1.14E-01	1.74E-01	3.19E-01	4.84E-01	2.74E-01	7.47E-01
	Prediag1	1.07E-05	6.54E-05	3.90E-01	5.64E-01	6.83E-01	8.79E-01
	Prediag2	3.71E-01	4.75E-01	5.83E-01	6.16E-01	9.63E-01	9.85E-01
	Prediag3	1.25E-03	3.45E-03	3.28E-02	9.58E-02	7.65E-02	4.21E-01
	Prediag4	5.13E-01	5.73E-01	1.12E-01	2.47E-01	4.07E-02	3.55E-01
	Treat	7.43E-04	2.55E-03	1.60E-01	3.14E-01	1.73E-02	1.90E-01
**Fraction**	Postdiag	2.31E-01	3.18E-01	5.30E-02	1.39E-01	7.24E-01	8.79E-01
	Prediag1	2.40E-06	1.89E-05	4.76E-01	5.81E-01	7.01E-01	8.79E-01
	Prediag2	4.22E-01	5.16E-01	4.81E-01	5.81E-01	9.85E-01	9.85E-01
	Prediag3	1.12E-03	3.25E-03	3.48E-02	9.58E-02	7.47E-02	4.21E-01
	Prediag4	5.15E-01	5.73E-01	1.25E-01	2.65E-01	4.52E-02	3.55E-01
	Treat	5.69E-04	2.09E-03	1.68E-01	3.18E-01	1.38E-02	1.90E-01
**Peaktime**	Postdiag	5.21E-01	5.73E-01	7.57E-01	7.85E-01	3.34E-01	7.47E-01
	Prediag1	4.95E-01	5.73E-01	1.44E-01	2.93E-01	4.78E-01	8.79E-01
	Prediag2	3.10E-01	4.06E-01	2.50E-01	4.17E-01	6.59E-01	8.79E-01
	Prediag3	9.78E-02	1.58E-01	5.72E-01	6.16E-01	3.39E-01	7.47E-01
	Prediag4	4.73E-01	5.65E-01	1.89E-01	3.35E-01	9.78E-02	4.89E-01
	Treat	2.02E-02	4.28E-02	1.76E-01	3.23E-01	4.14E-01	8.44E-01

* From a false discovery rate of 0.05 from running a Wilcoxon Rank-Sum test on comparing populations that did and did not survive or relapse in a 5-year period for lung cancer patients.

Suppl. Table 3: The Significant Features Associated with Lung Cancer Patient Overall Survival and Relapse-Free Survival Derived from the Adjusted[1] LASSO Model

Feature *Lung*	Time Period	OR[2] (95th CI)	p - value
**Stages I/II Mortality**			
Max	Postdiag	1.4(1.2-1.6)	6.68E-06
Max	Prediag1	1.3(1.2-1.5)	4.10E-06
Max	Prediag3	1.2(1-1.3)	2.24E-02
Max	Prediag4	1.2(1.1-1.3)	3.87E-03
Min	Prediag1	0.73(0.65-0.81)	3.43E-08
Min	Prediag2	0.86(0.77-0.96)	7.21E-03
Min	Prediag3	0.85(0.76-0.95)	4.59E-03
Min	Prediag4	0.89(0.8-0.99)	3.19E-02
AG400	Prediag2	2.5(1.6-3.8)	1.96E-05
AG400	Prediag3	1.6(1.1-2.4)	2.25E-02
Freq	Postdiag	1.2(1-1.3)	1.24E-02
Freq	Prediag3	0.86(0.76-0.98)	1.78E-02
Peak	Prediag4	0.93(0.88-0.98)	7.31E-03
Fraction	Prediag3	1.1(1-1.2)	1.98E-03
Age	Overall	1(1-1.1)	4.16E-43
Tobacco – Current[4]	Overall	2(1.5-2.8)	1.96E-05
Tobacco – Previous[4]	Overall	1.6(1.1-2.2)	5.43E-03
Race – Black[5]	Overall	1.6(1.2-2.3)	4.41E-03
**Stages III/IV Mortality**			
Min	Prediag1	0.8(0.72-0.89)	6.84E-05
Age	Overall	1(1-1)	3.91E-17
Tobacco – Current[4]	Overall	1.6(1.1-2.3)	1.02E-02
Tobacco – Unknown[4]	Overall	1.6(1.1-2.4)	2.06E-02
**Stages I/II Recurrence**			
Age	Overall	0.99(0.98-1)	1.59E-02
Race – Black[5]	Overall	0.76(0.59-0.98)	3.97E-02

[1] All of the features that were found to have p-values less than 0.05 in a logistic regression analysis adjusted for race, age, gender, and tobacco usage

[2] OR (odds ratio) derived from the exponentiation of the beta coefficient.

[3] Reference group is Gender – Male

[4] Reference group is Tobacco - Never

[5] Reference group is Race – White

.

Suppl. Table 4: The Significant Features Associated with Lung Cancer Patient Overall Survival and Relapse-Free Survival Derived from the Adjusted[1] Postdiag AG400 Model

**Feature** *Lung*	**Time Period**	**OR[2] (95th CI)**	**p - value**
**Stages I/II Mortality**			
AG400	Postdiag	1.6(1.3-1.9)	3.57E-06
Age	Overall	1(1-1.1)	2.84E-41
Gender – Female[3]	Overall	0.72(0.51-1)	4.95E-02
Tobacco – Current[4]	Overall	2(1.5-2.8)	1.36E-05
Tobacco – Previous[4]	Overall	1.5(1.1-2.1)	6.32E-03
Tobacco – Unknown[4]	Overall	1.6(1.2-2.2)	5.43E-03
Race – Black[4]	Overall	0.87(0.75-1)	4.30E-02
**Stages III/IV Mortality**			
AG400	Postdiag	1.6(1.3-1.9)	2.18E-07
Age	Overall	1(1-1)	1.08E-16
Tobacco – Current[4]	Overall	1.6(1.1-2.3)	1.63E-02
Tobacco – Unknown[4]	Overall	1.6(1-2.3)	2.70E-02
**Stages I/II Recurrence**			
Age	Overall	0.98(0.97-1)	5.29E-03
Tobacco – Current[4]	Overall	3.9(1.7-11)	3.38E-03
Tobacco – Previous[4]	Overall	3.7(1.7-11)	4.81E-03
Tobacco – Unknown[4]	Overall	3(1.3-8.8)	2.21E-02
Race – Black[5]	Overall	0.69(0.53-0.9)	6.32E-03

[1] All of the features that were found to have p-values less than 0.05 in a logistic regression analysis adjusted for race, age, gender, and tobacco usage

[2] OR (odds ratio) derived from the exponentiation of the beta coefficient.

[3] Reference group is Gender – Male

[4] Reference group is Tobacco - Never

[5] Reference group is Race – White

Suppl. Table 5: Summary Statistics of the Prostate Cancer Patients

Characteristics *Prostate Cancer*	Total n (% of 16,529)	# of plt counts median (25th- 75th percentile)	5-Year OS n (% of Total Column)	Mean Platelet Count (± SD)	p-value (from mean platelet count)
**Overall**	16,529 (100%)	14 (10 - 20)	12,510 (75%)	229±86	
**Age(years)**					
**< 60**	2,474(15%)	13 (10 - 19)	2,245 (91%)	240±63	
**≥ 60**	14,055 (85%)	14 (10 - 20)	10,265 (73%)	226±63	P < 0.0001
**Cancer stage**					
**I/II**	3,663 (78%)	14 (10 - 19)	10,549 (82%)	228±66	
**III/IV**	12,866 (22%)	15 (11 - 20)	1,961 (54%)	232±62	P = 0.001
**Race**					
**Black**	4,844 (29%)	15 (11 - 21)	3,714 (77%)	233±65	
**White**	11,332 (69%)	14 (10 - 19)	8,512 (75%)	227±62	
**Asian**	64 (0.04%)	14 (10 - 21)	54 (84%)	216±65	P < 0.0001
**Other**	289 (1.7%)	12 (9 - 17)	230 (80%)	232±63	
**Smoking**					
**Never**	3,312 (20%)	13 (10 - 19)	2,742 (83%)	220±59	
**Previously**	5,597 (34%)	14 (10 - 20)	4,145 (74%)	226±63	
**Current**	4,537 (27%)	14 (10 - 20)	3,355 (74%)	236±65	< 0.0001
**Unknown**	3,083 (19%)	15 (11 - 21)	922 (74%)	231±64	
**Treatment***					
**Surgery**	6,506 (40%)	13 (10 - 18)	5,564 (86%)	234±59	
**Chemotherapy**	1,489 (9%)	18 (14 - 24)	604 (41%)	233±66	
**Radiation**	5,926 (36%)	15 (11 - 20)	2,466 (25%)	226±63	P = 0.02
**Immunotherapy**	111 (0.06%)	17 (13 - 24)	77 (69%)	229±57	
**Unknown**	2,282 (14%)	15 (11 - 21)	1,659 (73%)	226±64	

Suppl. Table 6: Summary Statistics of the Colon Cancer Patients

Characteristics *Colon Cancer*	Total n (% of 6,050)	# of plt counts median (25th- 75th percentile)	5-Year OS n (% of Total Column)	Mean Platelet Count (± SD)	p-value (from mean platelet count)
**Overall**	6,050 (100%)	16 (12 - 22)	3,527 (58%)	237±79	
**Age(years)**					
**< 60**	620 (10%)	16 (12 - 22)	429 (69%)	252±71	
**≥ 60**	5,430 (90%)	15 (12 - 21)	3,098 (57%)	235±67	P < 0.0001
**Gender**					
**Male**	5,904 (98%)	15 (12 - 22)	3,426 (58%)	236±67	
**Female**	146 (2%)	14 (11 - 19)	101 (69%)	272±74	P < 0.0001
**Cancer stage**					
**I/II**	3,559 (59%)	17 (13 - 22)	1,093 (44%)	235±67	
**III/IV**	2,491 (41%)	15 (11 - 21)	2,434 (68%)	239±68	P = 0.04
**Race**					
**Black**	1,076 (18%)	16 (10 - 19)	618 (57%)	247±69	
**White**	4,844 (80%)	16 (12 - 22)	2,831 (58%)	234±67	
**Asian**	31 (0.05%)	14 (10 - 21)	19 (61%)	253±74	P < 0.0001
**Other**	99 (1.6%)	15 (12 - 22)	59 (60%)	241±75	
**Smoking**					
**Never**	1,139 (19%)	15 (12 - 20)	716 (63%)	235±64	
**Previously**	2,546 (42%)	16 (12 - 22)	1,426 (56%)	233±68	
**Current**	1,272 (21%)	16 (12 - 21)	749 (59%)	244±68	< 0.0001
**Unknown**	1,093 (18%)	16 (12 - 23)	636 (58%)	239±69	
**Treatment***					
**Surgery**	4,740 (78%)	15 (11 - 21)	3,061 (65%)	237±67	
**Chemotherapy**	2,037 (34%)	17 (13 - 23)	1,045 (51%)	238±67	
**Radiation**	521 (8.6%)	18 (14 - 23)	291 (56%)	237±69	P = 0.94
**Immunotherapy**	94 (1.6%)	19 (13 - 24)	41 (44%)	239±71	
**Unknown**	350 (5.8%)	17 (12 - 24)	118 (34%)	237±72	

Suppl. Table 7: Wilcoxon Rank Sum Test P-values and Adjusted P-values* in Prostate Cancer Patients

		Prostate Stage I/II Survival		Prostate Stage III/IV Survival		Prostate Stage I/II Relapse-Free Survival	
Variables	**Period**	**P-value**	**Adjusted P-value**	**P-value**	**Adjusted P-value**	**P-value**	**Adjusted P-value**
**Max**	Postdiag	1.60E-01	2.10E-01	1.16E-13	1.27E-12	8.07E-01	9.89E-01
	Prediag1	1.06E-02	1.53E-02	5.39E-10	3.62E-09	9.53E-01	9.89E-01
	Prediag2	6.74E-01	7.56E-01	6.30E-01	6.54E-01	9.98E-01	9.98E-01
	Prediag3	4.80E-01	5.86E-01	5.19E-01	5.94E-01	8.78E-01	9.89E-01
	Prediag4	4.69E-01	5.86E-01	1.98E-01	2.54E-01	7.78E-01	9.89E-01
	Treat	8.82E-02	1.21E-01	1.59E-03	4.37E-03	6.49E-03	1.51E-01
**Min**	Postdiag	5.78E-02	8.16E-02	4.30E-10	3.38E-09	8.58E-01	9.89E-01
	Prediag1	3.17E-21	5.82E-20	6.51E-05	2.24E-04	3.33E-01	9.89E-01
	Prediag2	1.72E-20	2.37E-19	1.02E-06	5.08E-06	2.51E-01	9.89E-01
	Prediag3	1.97E-13	2.17E-12	4.20E-06	1.78E-05	6.55E-01	9.89E-01
	Prediag4	2.33E-09	1.28E-08	7.44E-06	2.91E-05	7.30E-01	9.89E-01
	Treat	3.97E-24	1.09E-22	9.54E-14	1.27E-12	9.44E-02	5.36E-01
**AG400**	Postdiag	4.89E-09	2.24E-08	2.70E-22	4.95E-21	6.66E-01	9.89E-01
	Prediag1	7.19E-06	1.88E-05	7.00E-10	3.85E-09	9.19E-01	9.89E-01
	Prediag2	1.97E-03	3.09E-03	5.87E-02	8.28E-02	1.76E-01	8.05E-01
	Prediag3	2.03E-04	3.75E-04	8.24E-03	1.56E-02	9.75E-02	5.36E-01
	Prediag4	1.65E-05	3.95E-05	4.71E-02	7.00E-02	7.45E-01	9.89E-01
	Treat	4.99E-12	3.92E-11	7.94E-06	2.91E-05	8.49E-01	9.89E-01
**AG800**	Postdiag	7.98E-01	8.28E-01	4.83E-01	5.65E-01	6.21E-01	9.89E-01
	Prediag1	6.34E-01	7.32E-01	1.85E-01	2.42E-01	3.90E-01	9.89E-01
	Prediag2	9.88E-01	9.88E-01	5.44E-01	5.98E-01	5.35E-01	9.89E-01
	Prediag3	9.32E-01	9.49E-01	9.20E-01	9.20E-01	6.47E-01	9.89E-01
	Prediag4	1.51E-01	2.03E-01	3.08E-03	8.07E-03	5.97E-01	9.89E-01
	Treat	7.42E-01	7.91E-01	1.86E-02	3.19E-02	5.75E-01	9.89E-01
**Freq**	Postdiag	4.61E-09	2.24E-08	2.33E-22	4.95E-21	6.64E-01	9.89E-01
	Prediag1	5.43E-06	1.58E-05	5.93E-10	3.62E-09	9.00E-01	9.89E-01
	Prediag2	2.11E-03	3.23E-03	5.47E-02	7.92E-02	1.71E-01	8.05E-01
	Prediag3	2.05E-04	3.75E-04	7.23E-03	1.42E-02	9.62E-02	5.36E-01
	Prediag4	1.33E-05	3.31E-05	4.47E-02	6.83E-02	7.67E-01	9.89E-01
	Treat	2.10E-12	1.93E-11	3.31E-06	1.52E-05	9.07E-01	9.89E-01
**Peak**	Postdiag	4.35E-06	1.50E-05	3.64E-04	1.11E-03	9.74E-01	9.92E-01
	Prediag1	1.72E-04	3.47E-04	6.03E-03	1.23E-02	4.01E-01	9.89E-01
	Prediag2	7.17E-04	1.23E-03	4.03E-02	6.34E-02	8.64E-01	9.89E-01
	Prediag3	5.71E-08	2.24E-07	5.05E-03	1.11E-02	6.72E-02	5.28E-01
	Prediag4	8.65E-11	5.94E-10	5.93E-01	6.27E-01	8.88E-01	9.89E-01
	Treat	6.81E-06	1.87E-05	4.25E-01	5.19E-01	8.25E-03	1.51E-01
**Fraction**	Postdiag	4.26E-06	1.50E-05	1.81E-04	5.86E-04	8.31E-01	9.89E-01
	Prediag1	1.26E-04	2.66E-04	3.44E-03	8.60E-03	3.91E-01	9.89E-01
	Prediag2	6.56E-04	1.16E-03	3.02E-02	5.04E-02	9.00E-01	9.89E-01
	Prediag3	2.11E-08	8.91E-08	5.01E-03	1.11E-02	6.32E-02	5.28E-01
	Prediag4	9.81E-11	5.99E-10	6.73E-01	6.85E-01	8.98E-01	9.89E-01
	Treat	5.44E-06	1.58E-05	5.35E-01	5.98E-01	1.28E-02	1.76E-01
**Peaktime**	Postdiag	1.77E-04	3.47E-04	4.83E-01	5.65E-01	4.40E-01	9.89E-01
	Prediag1	5.69E-01	6.80E-01	1.85E-02	3.19E-02	9.36E-01	9.89E-01
	Prediag2	7.28E-01	7.91E-01	1.43E-01	1.92E-01	3.40E-01	9.89E-01
	Prediag3	6.38E-01	7.32E-01	2.91E-01	3.64E-01	3.22E-01	9.89E-01
	Prediag4	4.36E-01	5.57E-01	5.72E-01	6.17E-01	8.63E-01	9.89E-01
	Treat	7.65E-05	1.68E-04	1.49E-03	4.32E-03	9.25E-01	9.89E-01

* From a false discovery rate of 0.05 from running a Wilcoxon Rank-Sum test on comparing populations that did and did not survive or relapse in a 5-year period

Suppl. Table 8: Wilcoxon Rank Sum Test P-values and Adjusted P-values* in Colon Cancer Patients

		Colon Stage I/II Survival		Colon Stage III/IV Survival		Colon Stage I/II Relapse-Free Survival	
Variables	**Period**	**P-value**	**Adjusted P-value**	**P-value**	**Adjusted P-value**	**P-value**	**Adjusted P-value**
**Max**	Postdiag	5.70E-01	6.47E-01	1.28E-07	1.72E-06	6.42E-01	8.42E-01
	Prediag1	6.18E-01	6.67E-01	1.60E-06	1.44E-05	9.54E-02	5.25E-01
	Prediag2	6.52E-01	6.76E-01	6.88E-01	7.59E-01	1.17E-01	5.27E-01
	Prediag3	6.51E-01	6.76E-01	9.05E-01	9.05E-01	4.96E-01	7.58E-01
	Prediag4	5.59E-01	6.47E-01	4.33E-01	5.20E-01	2.53E-01	6.96E-01
	Treat	3.66E-01	4.57E-01	6.00E-04	2.70E-03	2.40E-01	6.95E-01
**Min**	Postdiag	1.05E-02	2.41E-02	6.89E-05	3.38E-04	3.09E-01	7.58E-01
	Prediag1	1.21E-06	1.34E-05	5.85E-01	6.58E-01	2.67E-01	6.98E-01
	Prediag2	1.86E-07	5.12E-06	7.49E-03	2.54E-02	3.45E-01	7.58E-01
	Prediag3	5.61E-05	3.43E-04	4.74E-02	9.84E-02	3.39E-01	7.58E-01
	Prediag4	4.36E-03	1.56E-02	4.26E-02	9.19E-02	5.51E-01	7.77E-01
	Treat	3.33E-06	3.05E-05	4.91E-01	5.64E-01	6.58E-01	8.42E-01
**AG400**	Postdiag	7.52E-03	1.88E-02	3.84E-06	2.59E-05	4.80E-01	7.58E-01
	Prediag1	6.00E-03	1.65E-02	1.49E-05	8.03E-05	2.96E-02	2.72E-01
	Prediag2	7.22E-04	3.61E-03	1.76E-02	4.53E-02	1.44E-01	5.27E-01
	Prediag3	2.65E-02	4.97E-02	2.39E-03	9.22E-03	6.70E-01	8.42E-01
	Prediag4	9.31E-01	9.31E-01	8.01E-03	2.54E-02	4.84E-01	7.58E-01
	Treat	7.19E-03	1.88E-02	2.36E-08	4.25E-07	1.93E-01	6.24E-01
**AG800**	Postdiag	1.92E-01	2.77E-01	3.77E-01	4.90E-01	1.72E-02	2.37E-01
	Prediag1	4.97E-01	5.94E-01	1.26E-01	2.12E-01	8.28E-01	8.68E-01
	Prediag2	5.76E-01	6.47E-01	1.26E-01	2.12E-01	7.57E-01	8.68E-01
	Prediag3	3.75E-02	6.25E-02	3.77E-01	4.90E-01	7.57E-01	8.68E-01
	Prediag4	1.41E-01	2.10E-01	3.77E-01	4.90E-01	8.28E-01	8.68E-01
	Treat	2.06E-03	9.45E-03	8.77E-01	8.94E-01	2.19E-01	6.70E-01
**Freq**	Postdiag	5.20E-03	1.56E-02	3.66E-06	2.59E-05	4.45E-01	7.58E-01
	Prediag1	4.89E-03	1.56E-02	1.08E-05	6.51E-05	2.72E-02	2.72E-01
	Prediag2	7.01E-04	3.61E-03	1.53E-02	4.13E-02	1.44E-01	5.27E-01
	Prediag3	2.82E-02	5.00E-02	2.13E-03	8.86E-03	6.74E-01	8.42E-01
	Prediag4	9.03E-01	9.20E-01	7.89E-03	2.54E-02	4.69E-01	7.58E-01
	Treat	3.90E-03	1.53E-02	2.05E-08	4.25E-07	1.92E-01	6.24E-01
**Peak**	Postdiag	2.27E-01	3.12E-01	2.92E-01	4.26E-01	6.24E-01	8.42E-01
	Prediag1	8.23E-06	6.47E-05	1.65E-01	2.63E-01	3.66E-01	7.58E-01
	Prediag2	1.11E-02	2.45E-02	7.78E-02	1.45E-01	6.72E-02	5.25E-01
	Prediag3	4.41E-07	6.32E-06	3.33E-01	4.73E-01	8.37E-01	8.68E-01
	Prediag4	5.37E-03	1.56E-02	4.22E-01	5.18E-01	9.67E-01	9.67E-01
	Treat	2.71E-02	4.97E-02	2.86E-02	6.44E-02	1.02E-05	1.87E-04
**Fraction**	Postdiag	3.22E-01	4.12E-01	3.81E-01	4.90E-01	8.14E-01	8.68E-01
	Prediag1	2.26E-05	1.55E-04	1.53E-01	2.51E-01	4.24E-01	7.58E-01
	Prediag2	2.21E-02	4.35E-02	5.49E-02	1.09E-01	8.09E-02	5.25E-01
	Prediag3	4.60E-07	6.32E-06	3.43E-01	4.75E-01	8.22E-01	8.68E-01
	Prediag4	4.65E-03	1.56E-02	4.21E-01	5.18E-01	9.51E-01	9.67E-01
	Treat	1.48E-02	3.14E-02	1.41E-02	4.00E-02	8.90E-06	1.87E-04
**Peaktime**	Postdiag	3.75E-02	6.25E-02	8.62E-01	8.94E-01	7.57E-01	8.68E-01
	Prediag1	8.96E-03	2.14E-02	8.52E-02	1.53E-01	4.88E-01	7.58E-01
	Prediag2	2.08E-01	2.94E-01	5.66E-02	1.09E-01	3.95E-01	7.58E-01
	Prediag3	6.95E-02	1.09E-01	2.81E-01	4.22E-01	4.69E-01	7.58E-01
	Prediag4	2.63E-01	3.52E-01	7.63E-01	8.08E-01	5.35E-01	7.75E-01
	Treat	6.08E-01	6.67E-01	2.27E-02	5.34E-02	8.85E-02	5.25E-01

* From a false discovery rate of 0.05 from running a Wilcoxon Rank-Sum test on comparing populations that did and did not survive or relapse in a 5-year period

Suppl. Table 9: The Significant Features Associated with Prostate Cancer Patient Overall Survival and Relapse-Free Survival Derived from the Adjusted[1] LASSO Model

Feature *Prostate*	Time Period	OR[2] (95th CI)	p - value
**Stages I/II Mortality**			
Max	Postdiag	1.4(1.2-1.6)	1.77E-06
Max	Prediag1	1.3(1.2-1.5)	3.04E-06
Max	Prediag2	1.3(1.1-1.4)	5.36E-04
Max	Prediag4	1.2(1.1-1.4)	4.22E-03
Min	Postdiag	0.86(0.77-0.97)	1.24E-02
Min	Prediag1	0.72(0.63-0.82)	2.85E-07
Min	Prediag2	0.63(0.55-0.72)	9.29E-12
Peak	Postdiag	1.1(1-1.2)	3.16E-02
Peak	Prediag3	1.1(1-1.2)	1.24E-02
Fraction	Prediag4	1.1(1-1.2)	7.61E-04
Peaktime	Prediag3	1.1(1.1-1.3)	2.82E-03
Peaktime	Prediag4	1.1(1-1.1)	4.78E-02
Age	Overall	1.1(1.1-1.1)	2.27E-114
Tobacco – Current[4]	Overall	2.6(2.2-3.2)	1.41E-23
Tobacco – Previous[4]	Overall	1.7(1.4-2.1)	4.69E-09
Tobacco – Unknown[4]	Overall	1.7(1.4-2.1)	1.05E-07
**Stages III/IV Mortality**			
Max	Postdiag	1.4(1.1-1.9)	5.85E-03
Min	Prediag1	0.46(0.28-0.75)	2.04E-03
Min	Prediag2	0.7(0.56-0.87)	1.15E-03
Min	Prediag3	0.74(0.59-0.93)	8.66E-03
Fraction	Prediag3	1.2(1.1-1.5)	1.27E-02
Peaktime	Prediag1	0.84(0.68-0.96)	3.40E-02
Peaktime	Prediag4	0.85(0.7-0.99)	4.83E-02
Age	Overall	1.1(1.1-1.1)	2.27E-114
Tobacco – Current[4]	Overall	2.6(2.2-3.2)	1.41E-23
Tobacco – Previous[4]	Overall	1.7(1.4-2.1)	4.69E-09
Tobacco – Unknown[4]	Overall	1.7(1.4-2.1)	1.05E-07
**Stages I/II Recurrence**			
Age	Overall	1(1-1)	2.07E-02
Tobacco – Current[4]	Overall	1.8(1.2-2.7)	5.40E-03
Tobacco – Previous[4]	Overall	1.5(1-2.3)	4.81E-02
Race – Black[5]	Overall	0.73(0.53-0.99)	4.66E-02

[1] All of the features that were found to have p-values less than 0.05 in a logistic regression analysis adjusted for race, age, gender, and tobacco usage

[2] OR (odds ratio) derived from the exponentiation of the beta coefficient.

[3] Reference group is Gender – Male

[4] Reference group is Tobacco - Never

[5] Reference group is Race – White

Suppl. Table 10: The Significant Features Associated with Colon Cancer Patient Survival and Relapse-Free Survival Derived from the Adjusted[1] LASSO Model

Feature *Colon*	Time Period	OR[2] (95th CI)	p - value
**Stages I/II Mortality**			
Max	Postdiag	1.5(1.2-2)	1.51E-03
Max	Prediag1	1.9(1.1-3.4)	2.24E-02
Max	Prediag4	1.6(1.3-1.9)	1.48E-06
Min	Prediag2	0.62(0.49-0.79)	6.58E-05
AG400	Prediag2	3.4(1.4-8.4)	5.60E-03
Peak	Postdiag	0.79(0.66-0.94)	8.71E-03
Peak	Prediag1	1.4(1.1-1.8)	2.10E-02
Peak	Prediag2	0.54(0.33-0.77)	3.90E-03
Peak	Prediag4	0.64(0.46-0.86)	5.20E-03
Fraction	Prediag3	1.2(1.1-1.4)	1.32E-04
Fraction	Prediag4	1.4(1.2-1.8)	1.34E-03
Peaktime	Prediag2	0.73(0.6-0.86)	4.58E-04
Age	Overall	1.1(1.1-1.1)	3.07E-31
**Stages III/IV Mortality**			
Max	Prediag1	1.4(1.1-1.8)	9.88E-03
Min	Prediag2	0.67(0.53-0.84)	4.96E-04
Age	Overall	1(1-1.1)	1.42E-11
**Stages I/II Recurrence**			
None			

[1] All of the features that were found to have p-values less than 0.05 in a logistic regression analysis adjusted for race, age, gender, and tobacco usage

[2] OR (odds ratio) derived from the exponentiation of the beta coefficient.

[3] Reference group is Gender – Male

[4] Reference group is Tobacco - Never

[5] Reference group is Race – White

Suppl. Table 11: The Significant Features Associated with Prostate and Colon Cancer Patient Overall Survival and Relapse-Free Survival Derived from the Adjusted[1] Postdiag AG400 Model

Feature *Prostate*	Time Period	OR[2] (95th CI)	p - value
**Stages I/II Mortality**			
AG400	Postdiag	1.7(1.3-2.2)	2.92E-05
Age	Overall	1.1(1.1-1.1)	4.72E-124
Tobacco – Current[4]	Overall	2.8(2.3-3.4)	3.56E-27
Tobacco – Previous[4]	Overall	1.8(1.5-2.1)	5.71E-10
Tobacco – Unknown[4]	Overall	1.8(1.5-2.2)	3.59E-09
Race – Black[5]	Overall	1.2(1.1-1.4)	4.04E-03
**Stages III/IV Mortality**			
AG400	Postdiag	3.7(2.6-5.3)	8.07E-13
Age	Overall	1.1(1.1-1.1)	8.21E-79
Tobacco – Current[4]	Overall	2.1(1.6-2.8)	1.37E-07
Tobacco – Previous[4]	Overall	1.7(1.3-2.3)	5.50E-05
Tobacco – Unknown[4]	Overall	2.1(1.6-2.9)	1.59E-06
Race – Black[5]	Overall	1.3(1-1.6)	1.97E-02
**Stages I/II Recurrence**			
Tobacco – Previous[4]	Overall	1.5(1.1-2.3)	2.91E-02
Tobacco – Unknown[4]	Overall	1.6(1-2.5)	3.41E-02

Feature *Colon*	Time Period	OR[1] (95th CI)	p - value
**Stages I/II Mortality**			
AG400	Postdiag	1.6(1.2-2.2)	3.71E-03
Age	Overall	1.1(1.1-1.1)	2.77E-31
Tobacco – Current[4]	Overall	1.5(1.1-2)	8.15E-03
Tobacco – Previous[4]	Overall	1.3(1-1.7)	3.22E-02
**Stages III/IV Mortality**			
AG400	Postdiag	1.8(1.3-2.4)	6.21E-04
Age	Overall	1(1-1.1)	1.88E-13
Gender – Female[3]	Overall	0.53(0.28-0.98)	4.56E-02
Tobacco – Unknown[4]	Overall	1.4(1-2)	3.61E-02
**Stages I/II Recurrence**			
None			

[1] All of the features that were found to have p-values less than 0.05 in a logistic regression analysis adjusted for race, age, gender, and tobacco usage

[2] OR (odds ratio) derived from the exponentiation of the beta coefficient.

[3] Reference group is Gender – Male

[4] Reference group is Tobacco - Never

[5] Reference group is Race – White
